# CCL7 contributes to angiotensin II‐induced abdominal aortic aneurysm by promoting macrophage infiltration and pro‐inflammatory phenotype

**DOI:** 10.1111/jcmm.16757

**Published:** 2021-06-29

**Authors:** Cuiping Xie, Feiming Ye, Ning Zhang, Yuxue Huang, Yun Pan, Xiaojie Xie

**Affiliations:** ^1^ Department of Cardiology Cardiovascular Key Laboratory of Zhejiang Province Zhejiang University School of Medicine Second Affiliated Hospital Hangzhou China; ^2^ College of Information Science and Electronic Engineering Zhejiang University Hangzhou China

**Keywords:** abdominal aortic aneurysm, angiotensin II, Chemokine C‐C motif ligand 7, M1 phenotype, macrophage

## Abstract

Chemokine C‐C motif ligand 7 (CCL7), a member of CC chemokine subfamily, plays pivotal roles in numerous inflammatory diseases. Hyper‐activation of inflammation is an important characteristic of abdominal aortic aneurysm (AAA). Therefore, in the present study, we aimed to determine the effect of CCL7 on AAA formation. CCL7 abundance in aortic tissue and macrophage infiltration were both increased in angiotensin II (Ang II)‐induced AAA mice. Ex vivo, CCL7 promoted macrophage polarization towards M1 phenotype. This effect was reversed by the blockage of CCR1, a receptor of CCL7. CCL7 up‐regulated JAK2/STAT1 protein level in macrophage, and CCL7‐induced M1 activation was suppressed by JAK2/STAT1 pathway inhibition. To verify the effect of CCL7 on AAA in vivo, either CCL7‐neutralizing antibody (CCL7‐nAb) or vehicles were intraperitoneally injected 24 hours prior to Ang II infusion and subsequently every three days for 4 weeks. CCL7‐nAb administration significantly attenuated Ang II‐induced luminal and external dilation as well as pathological remodelling. Immunostaining showed that CCL7‐nAb administration significantly decreased aneurysmal macrophage infiltration. In conclusion, CCL7 contributed to Ang II‐induced AAA by promoting M1 phenotype of macrophage through CCR1/JAK2/STAT1 signalling pathway.

## 1 INTRODUCTION

Abdominal aortic aneurysm (AAA) is a chronic vascular disease characterized by progressive and irreversible luminal dilation of the abdominal aorta. It eventually results in aortic rupture without appropriate clinical intervention which is of extremely high mortality.^1^, ^2^ Most patients are asymptomatic until the aneurysms get ruptured. Unfortunately, there are no effective drugs to prevent or reverse the progression of the disease so far. Therefore, it is an urgent requirement to understand the mechanisms and identify therapeutic targets of AAA.

There has been compelling evidence that inflammation plays a pivotal role in AAA. It is generally accepted that AAA is essentially a chronic inflammatory disease. Endothelium damage is the initial step of AAA formation. Then, circulating monocytes infiltrate into the aortic wall and transform to macrophages which become the primary source of pro‐inflammatory mediators and oxidative stress, provocating vascular inflammation and remodelling.^3^ Basically, two major macrophage subsets are involved in AAA development. M1, the pro‐inflammatory phenotype, is responsible for synthesizing pro‐inflammatory molecules and matrix metalloproteinases, thus promoting the formation of AAA. M2, on the contrary, exerts anti‐inflammatory effects and contributes to tissue repair. Its protective property has been demonstrated in the development of AAA.^3^, ^4^

Chemokine C‐C motif ligand 7 (CCL7), also known as monocyte‐specific chemokine 3 (MCP‐3), is a member of the C‐C chemokine ligand family. CCL7 has been confirmed to mediate monocyte/macrophage mobilization.^5^, ^6^ It is reported that deletion of CCL7 dramatically reduced monocyte recruitment to inflammatory site.^7^ CCL7 attracts monocyte/macrophage through binding to its specific surface receptors CCRs, including CCR1, CCR2, CCR3 and CCR5. The involvement of CCL7‐CCR interaction has been recognized in multiple cardiovascular diseases (eg myocardial infarction and atherosclerosis).^8^ However, its role in AAA is still unknown.

In this study, we found that CCL7 promoted the M1 polarization of macrophage. Janus kinase (JAK)–signal transducer and activator of transcription (STAT) signalling is the major signal contributes to macrophage polarization. Previous studies have confirmed that STAT1 is a key mediator of M1 polarization while STAT6 is required to drive M2 polarization.^9^ Studies with macrophages show that deficiency of STAT1 or inhibition of JAK2/STAT1 pathway suppressed the expression of M1‐associated genes.^10^, ^11^, ^12^ Hence, we doubted that the mechanism of CCL7 on M1 polarization might be associated with JAK2/STAT1 regulation. In line with our speculation, CCL7 up‐regulated JAK2/STAT1 level in macrophage, and inhibition of JAK2/STAT1 pathway remarkably interrupted CCL7‐induced M1 polarization.

Our study revealed potential actions of CCL7 in AAA formation which was accomplished via promoting macrophage migration and transformation towards pro‐inflammatory phenotype (M1 phenotype) through JAK2/STAT1 pathway.

## 2 MATERIALS AND METHODS

### 2.1 Mice

All animal studies were performed at the Cardiovascular Key Laboratory of Zhejiang Province, Second Affiliated Hospital, with the approval of Zhejiang University Institutional Animal Care and Use Committee. Male C57Bl/6 mice and apolipoprotein E‐deficient (*ApoE* ^‐/‐^) mice on C57BL/6 background were purchased from Shanghai SLAC Laboratory Animal Company and Model Animal Research Center of Nanjing University, respectively. Mice were bred in house under specific pathogen‐free conditions with free access to a normal chow diet and water, at a constant temperature (22 ± 2ºC) and humidity (60%–65%) with a 12 hour dark/light cycle.

### 2.2 Blood pressure measurement

Systolic blood pressure (SBP) was measured non‐invasively on conscious mice using a computerized tail‐cuff system (CODA 6+; Kent Scientific Corporation; Torrington) as described previously.^13^ All mice were acclimatized to the instrument for 7 days before the start of the study. Individual mouse received 5 initial pressure cycles to acclimatize the process and followed by 20 more cycles to get daily mean systolic blood pressure (SBP). The inclusion criteria of data were the acquisition of at least 10 of 20 measurements and an SD < 30 mmHg for each session.

### 2.3 Osmotic mini‐pump implantation and drug administration

Male *ApoE* ^‐/‐^ mice at 8‐10 weeks old were used in the study as described previously.^14^ Angiotensin II (Ang II) was purchased from Bachem (Cat# H1705; Torrance) and dissolved in sterile saline. Either Ang II (1000 ng/kg min) or saline was infused subcutaneously via osmotic mini‐pumps (ALZET Model 2004; Durect, Cupertino) for 4 weeks, respectively, as described previously.^15^ The osmotic mini‐pumps were subcutaneously implanted under general anaesthesia (inhalation by 4% isoflurane).

To investigate the role of CCL7 in Ang II‐induced AAA, *ApoE* ^‐/‐^ male mice were intraperitoneally injected with either CCL7‐neutralizing antibody (CCL7‐nAb, CAT# AF‐456‐NA, R&D SYSTEM) at a dose of 20 ug per mouse or vehicles 24 hours prior to Ang II infusion, and subsequently every three days for 4 weeks, this dosage decision was referred to previous researches.^16^, ^17^ Phosphate buffered saline (PBS) and control IgG antibody (normal goat IgG control, CAT# AB‐108‐C, R&D SYSTEM) were used as vehicles.

Mice were inspected daily and weighed weekly. Necropsy was performed for all mice that died prior to termination. Aortic rupture was defined as observation of blood clots in either the thoracic cavity (thoracic aortic rupture) or retroperitoneal cavity (abdominal aortic rupture). At termination, mice were sacrificed by cervical dislocation during inhalative isoflurane anaesthesia.

### 2.4 Quantification of abdominal aortic dilation

Maximal dimensions and areas of suprarenal lumen were monitored at selected intervals (D 0 and D 28) by a high‐frequency ultrasound imaging system (Visualsonics Vevo 2100) as described previously.^18^ Incidence of AAA was defined by either (1) 50% or more increase of the maximal diameter in the suprarenal aortic region as compared to the baseline or (2) death due to abdominal aortic rupture.^19^

At termination, right atrium was cut open, and PBS was perfused through the left ventricle to remove blood in the aorta. Subsequently, aortas were dissected and placed in 10% neutrally buffered formalin. After fixation, periaortic adventitia was removed thoroughly. Maximal width of suprarenal aortas was measured ex vivo as a parameter for AAA quantification using Image‐Pro software (Media Cybernetics; MD). All the data were quantified by two observers that were blinded to the study design.

### 2.5 Plasma measurement and histological staining

Plasma CCL7 concentrations were measured with ELISA kits according to manufacturer's recommendation (Cat# ab205571; Abcam).

Abdominal aortas containing AAAs were paraffin embedded and then serially cross‐sectioned (4 μm thick per section) from the proximal to the distal as described previously.^19^ Elastin was identified in selected sections with Verhoeff's van Gieson staining (Cat# ab150667; Abcam) and collagen was identified with Masson trichrome staining (Cat# G1306; Solarbio). Macrophages in abdominal aortas were detected by immunostaining with rat monoclonal anti‐mouse CD68 (Cat# MCA1957GA; Bio‐Rad) incubated in 4ºC overnight, followed by secondary antibody Biotinylated Anti‐Rat IgG (H + L) (Cat#BA‐4001,VECTOR) for 30 minutes and VECTASTAIN ELITE ABC Reagent (Cat#H‐6100, VECTOR) for 30 minutes at room temperature, a peroxidase‐based ABC system (Cat# PK7100; Vector) and the red chromogen, AEC, were used to visualize the antigen‐antibody reaction. Immunofluorescence co‐staining was performed with CD68 and iNOS (Cat# ab15323; abcam) incubated in 4ºC overnight, followed by secondary antibody Goat anti‐Rat IgG H&L (Dylight® 550) (Cat# ab96888, abcam) and Dnk anti‐Rb IgG H&L (Dylight® 488) (Cat# ab150073, abcam) for 1 hour at room temperature, cell nucleus was stained with DAPI (Cat# P0131, Beyotime). Ten visual fields (magnification × 200 or 400) of every lesion section were randomly included to count the numbers of macrophages and get the average of cell numbers in lesions.

### 2.6 Cell culture and migration assay

Bone marrow‐derived monocytes were isolated from 6 to 8 week‐old C57BL/6 male mice and cultured in RPMI1640 medium with 10% foetal bovine serum (FBS, Hyclone) as described previously.^20^ Monocytes were induced by murine granulocyte colony‐stimulating factor (G‐CSF, 10 ng/ml; Cat# 500‐P64;PeproTech) to differentiate for 7 days before the subsequent experiments.

Bone marrow‐derived macrophages (BMDMS) at a density of 1 × 10^5^ were placed in a 5 µm pore transwell insert (Cat# 3421; Corning) to evaluate cell migration as described previously.^21^ BMDMS was incubated with recombinant murine CCL7 (rmCCL7, Cat# 250‐08; PeproTech) for 6 hours at the final concentration of 100 ng/ml. Pre‐incubation with CCR1 antagonist‐BX471 (10 μM) was adopted 1 hour prior to incubation with rmCCL7 at the final concentration of 100 ng/ml. Ten visual fields (magnification × 100) of every transwell with crystal violet staining for nuclei were randomly included to count the numbers of macrophages and get the average of cell numbers.

HUVECs (ATCC) were cultured in Dulbecco's modified Eagle medium (low glucose, Gibico) and 10% FBS, Hyclone. Vascular smooth muscle cells (VSMC) and adventitial fibroblasts were isolated from the abdominal aorta of adult male wild‐type (WT) mice (C57BL/6J strain; Shanghai SLAC Laboratory Animal Co., Ltd) and cultured in Dulbecco's modified Eagle medium (high glucose for VSMC, low glucose for fibroblasts, Gibico) supplemented with 20% FBS, Hyclone and antibiotics as previously described.^22^, ^23^ HUVECs, VSMCs, fibroblasts and BMDMs were cultured with Ang II (at final concentration of 100 nM, 1 μM) (Cat# H1705; Torrance) and recombinant murine TNF‐α (at final concentration of 10 ng/ml) (Cat# AF‐315‐01A; PeproTech) for 24 hours, respectively. Every cell experiment repeated independently more than three times.

### 2.7 Isolation of RNA and quantitative real‐time PCR

Total RNA was reversely transcribed with a cDNA Synthesis kit (Cat# RR037A; Takara Bio), and quantitative PCR (qPCR) was performed to quantify mRNA abundance using a SYBR Green PCR Premix (Cat# RR420A; Takara Bio) on an Applied Biosystem cycler. Data were analysed using ΔΔCt method and GAPDH as internal control. Primers used in this study are listed in the Table S.

### 2.8 Western blotting

Protein lysate samples were prepared from snap frozen aortas and cells in RIPA solution (Cat# P0013B; Beyotime) supplemented with protease inhibitor (Cat# 05892791001; Roche). Denatured protein lysates were resolved by 8% and 15% (wt/vol) SDS‐PAGE gels. After transfer, membranes were blocked in 5% (wt/vol) non‐fat dry milk diluted in PBS. Membranes were incubated with primary antibodies against iNOS (Cat# ab15323; Abcam), CD206 (Cat# ab64693; Abcam), CCR1 (Cat# MAB5986; R&D system), JAK2 (Cat# 3250s; CST), p‐JAK2 (Cat# 3771;CST), STAT1 (Cat# sc346; Santa Cruz), p‐STAT1 (Cat# 8826s; CST), GAPDH (Cat# KC‐5G5; KangChen bio‐tech) and tubulin (Cat# KC‐5T01; KangChen bio‐tech) overnight at 4ºC and subsequently incubated with horseradish peroxidase (HRP) conjugated secondary antibodies which were detected by enhanced chemiluminescence (Cat# WBKLS0500; Millipore). Immunoblots were analysed using ImageLab software (Bio‐Rad).

### 2.9 Statistical analysis

SigmaPlot Version 12.5 (Systat Software Inc) was used for statistical analyses. To compare continuous response variables between 2 groups, an unpaired two‐tailed Student's *t* test was applied for normally distributed variables that passed the equal variance test, and Mann‐Whitney U test was used for variables not passing either normality or equal variance test. To compare more than 2 groups, one‐way ANOVA and Holm‐Sidak method was performed for normally distributed variables that passed equal variance test and Kruskal‐Wallis 1 way ANOVA on Ranks with Dunn method for variables failed to pass normality or equal variance test, respectively. *P* < .05 was considered as statistically significant.

## 3 RESULTS

### 3.1 CCL7 was up‐regulated in Ang II‐induced AAA

**FIGURE 1:**
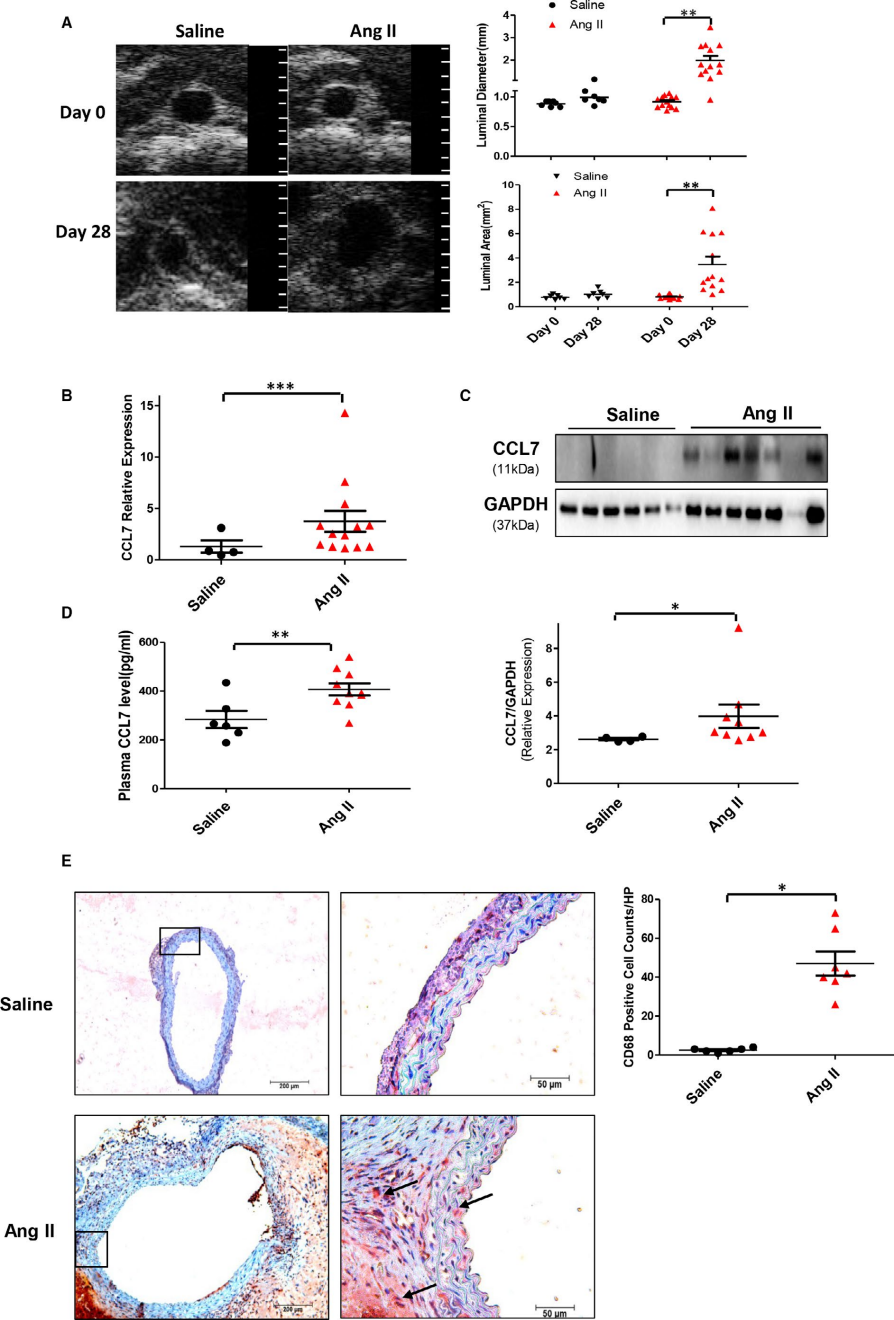
CCL7 expression and macrophages infiltration increased in Ang II‐infused abdominal aortas. (A) Representative abdominal aorta images with ultrasound examination, the maximum abdominal aorta diameter (luminal diameter) and the maximum abdominal aorta area (luminal area). A grid on the scale represented 0.5 mm. (B) qRT‐PCR for CCL7 mRNA expression in saline (n = 6) and Ang II (n = 7) infused mice. (C) Protein levels of CCL7 in saline (n = 6) and Ang II (n = 7) infused abdominal aortas. (D) Plasma concentrations of CCL7 in saline (n = 6) and Ang II (n = 9) infused mice. (E) Representative images of macrophage infiltration in aneurysmal lesions as revealed by CD68 immunostaining. Black arrows indicated CD68‐positive location, scale bars represented 200 μm (left) and 50 μm (right images). Values were represented as mean ± SEM; Student's *t* test was used for data analysis in (A‐E). *P < .05; **P < .01; ***P < .001, respectively

To investigate the involvement of CCL7 in AAA, we first generated Ang II‐induced AAA mouse model and measured the expression of CCL7. The success of AAA establishment was validated by ultrasound analysis at termination, which demonstrated a profound expansion of suprarenal aortic diameter and area of abdominal aortas in mice administered Ang II (Figure 1A). In Ang II‐infused group, 82.4% mice eventually survived and developed AAA, and the 17.6% mice were died of either abdominal aortic rupture or thoracic aortic rupture during the experiment. The expression of CCL7 in aortic tissues was strikingly increased in Ang II‐infused mice (Figures 1B,C). Consistently, plasma CCL7 concentration was also elevated in Ang II‐infused mice compared to saline group (Figure 1D). Macrophage‐mediated inflammation plays a crucial role in the pathogenesis of AAA. As expected, immunohistochemistry of CD68 revealed abundant macrophage accumulation in the medial and adventitial layers of aortic wall in Ang II‐infused group, whereas few macrophages were detected in control group (Figure 1E). Since both macrophage infiltration and CCL7 expression were increased in aneurysmal tissue, we hypothesized that CCL7 might be associated with macrophage biology in AAA development.

### 3.2 CCL7 promoted macrophage migration and M1 polarization through interaction with CCR1

**FIGURE 2:**
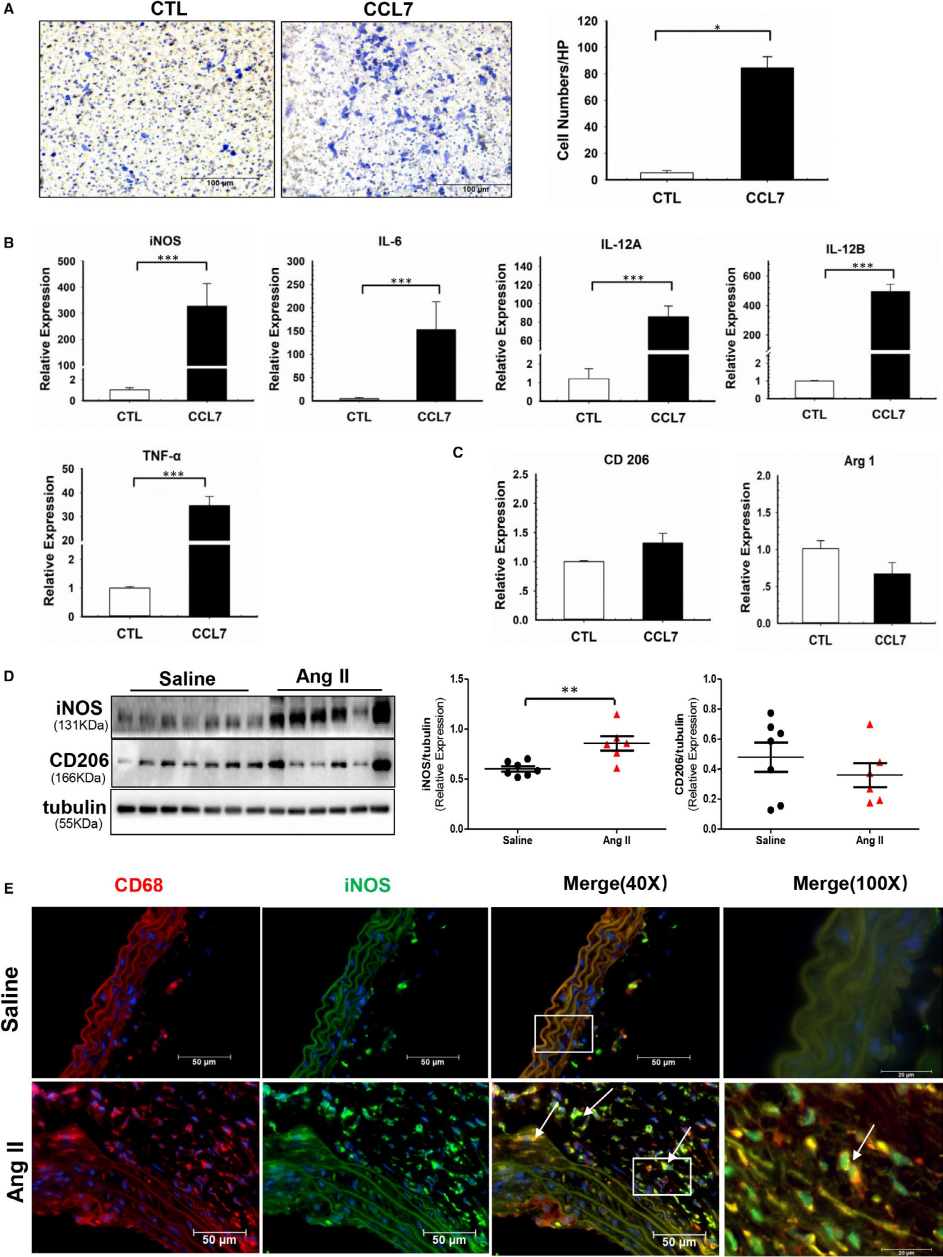
CCL7 promoted macrophages migration and M1 polarization. (A) Migration of BMDMs in response to rmCCL7(100 ng/ml) stimulation. Scale bars represented 100 μm(n = 3). (B and C), Expression of (B) M1 markers (iNOS, IL‐6, IL‐12A, IL‐12B, TNF‐α) and (C) M2 markers (Arg 1, CD206)in response to rmCCL7 in BMDMs examined by qRT‐PCR(n = 3). (D) Protein abundance of iNOS and CD206 in abdominal aortas of saline‐infused (n = 7) and Ang II‐infused (n = 6) mice. (E) Immunofluorescence co‐staining of CD68 and iNOS in abdominal aortas of saline‐infused (n = 3) and Ang II‐infused (n = 3) mice. Values were represented as mean ± SEM; Student's *t* test was used in (A‐D). *P < .05; **P < .01; ***P < .001, respectively

CCL7 predominantly acts as a chemokine that recruit macrophage into inflammatory site.^24^ Macrophage within the aortic wall exhibits diverse functions during vascular inflammation and remodelling. The role of the pro‐inflammatory M1 and the anti‐inflammatory M2 has been extensively acknowledged in the development and progression of AAA.^25^ To explore the regulatory role of CCL7 in macrophage, BMDMs were isolated from C57BL/6 mice and incubated with rmCCL7. Transwell experiment found that CCL7 promoted BMDMs migration (Figure 2A). To interpret whether CCL7 affected macrophage phenotype alteration, markers of M1 phenotype and M2 phenotype were examined. As a result, CCL7 led to a provocation of M1 as reflected by dramatic increase of iNOS, IL‐6, IL‐12A, IL‐12B and TNF‐α mRNA expression (Figure 2B), whereas mRNA level of Arg 1 and CD206 had no change (Figure 2C). Consistently, iNOS, IL‐6, IL‐12A, IL‐12B and TNF‐α mRNA expression was also increased in the aortic tissues of Ang II‐infused AAA mouse models at both mRNA and protein level (Figures S1,S2D, respectively), M1 macrophages remarkably increased in Ang II‐infused abdominal aortas indicated by immunofluorescence co‐staining of CD68 and iNOS (Figure 2E).

**FIGURE 3:**
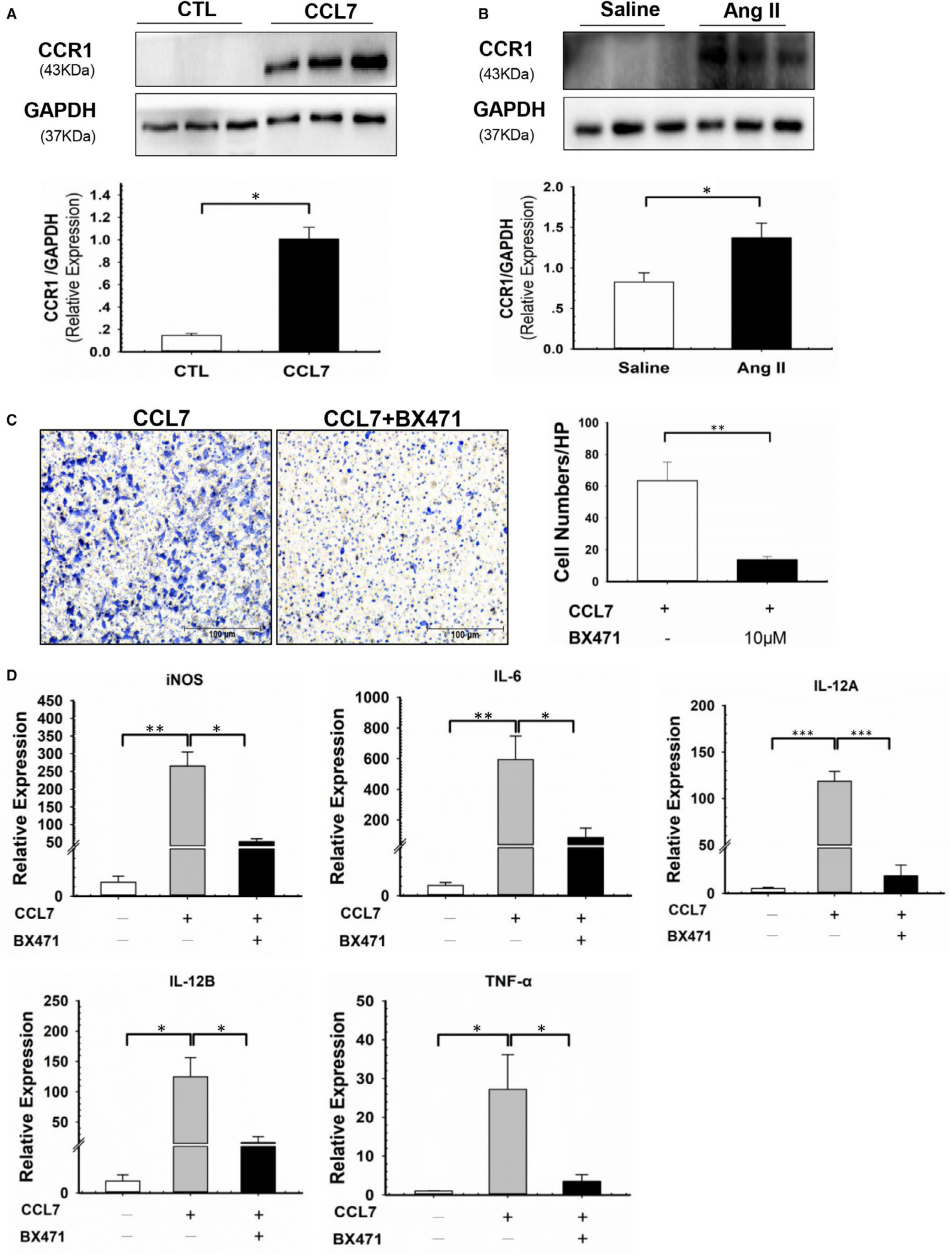
CCR1 antagonist‐BX471 suppressed CCL7‐induced macrophages migration and M1 polarization. (A and B) CCR1 protein level in (A) BMDMs incubated with rmCCL7(100 ng/ml) and (B) abdominal aortas of saline‐infused (n = 3) and Ang II‐infused (n = 3) mice. (C) Migration of BMDMs in response to rmCCL7 (100 ng/ml) and CCR1 antagonist‐BX471(10 μM) stimulation, BX471 was pre‐treated 1 hour before rmCCL7 incubation(n = 3). Scale bars represented 100 μm. (D) M1 markers (iNOS, IL‐6, IL‐12A, IL‐12B, TNF‐α) of BMDMs in response to rmCCL7 (100 ng/ml) and BX471(10 μM) stimulation, BX471 was pre‐treated 1 hour before rmCCL7 incubation(n = 3). Each plot represented 3 independent experiments. Values were represented as mean ± SEM; Student's *t* test was used in (A‐C). One‐way ANOVA was used for data analysis in (D). *P < .05; **P < .01; ***P < .001, respectively

Considering the biologic function of CCL7 is mediated by its crosstalk with CCRs, including CCR1, CCR2, CCR3 and CCR5,^26^ we next studied the receptor of CCL7 that participated in its regulation on macrophage function. Among these receptors, CCR1 was the only one that was significantly increased by CCL7 stimulation in BMDMs (Figure 3A, S2A). In accordance with ex vivo experiment, CCR1 expression in aortic tissues was also up‐regulated in Ang II‐infused AAA mice (Figure S2B,S3B). Therefore, we speculated that CCL7 might regulate macrophage function predominantly through interacting with CCR1. To address this hypothesis, BMDMs were pre‐treated with CCR1 antagonist, BX471, at final concentration of 10 μM for 1 hour and subsequently incubated with rmCCL7. Suppression of CCR1 significantly reduced CCL7‐induced macrophage migration and M1 polarization (Figure 3C,D). Thus, we concluded that CCL7 contributed to macrophage migration and M1 polarization via interacting with CCR1.

### 3.3 CCL7‐CCR1 interaction regulated macrophage phenotype through JAK2 /STAT1 pathway

**FIGURE 4:**
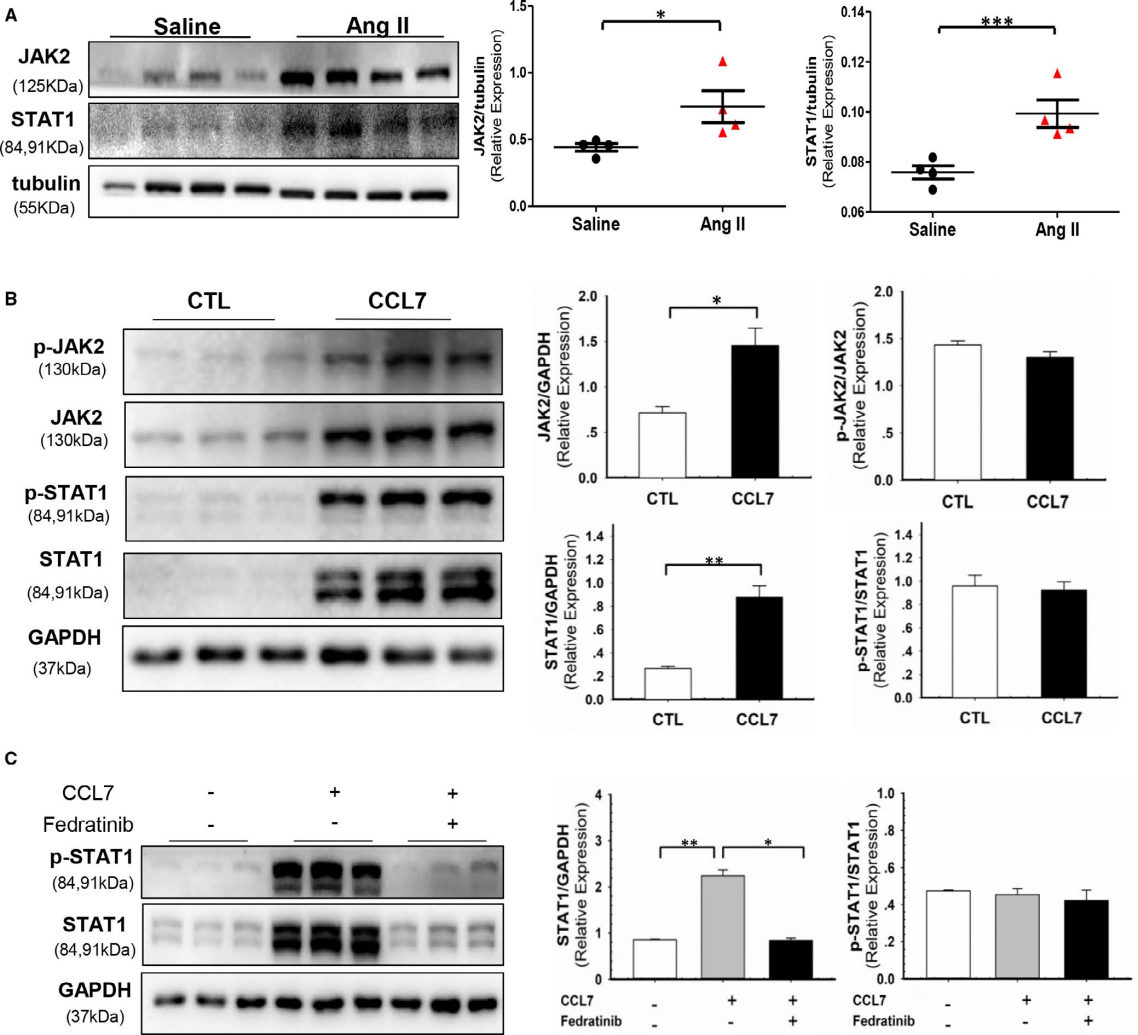
CCL7‐activated JAK2/STAT1 signal in macrophages. (A) Protein abundance of JAK2 and STAT1 in abdominal aortas of saline (n = 4) and Ang II‐infused (n = 4) mice. (B) Protein abundance of JAK2, p‐JAK2, STAT1, p‐STAT1 and p‐JAK2/JAK2, p‐STAT1/STAT1 ratio in BMDMs incubated with rmCCL7 (100 ng/ml). (C) Protein abundance of STAT1, p‐STAT1 and p‐STAT1/STAT1 ratio in BMDM incubated with rmCCL7(100 ng/ml) and pre‐treated 1 hour with JAK2 inhibitor Fedratinib (1 μM). Values were represented as mean ± SEM; Student's *t* test was used for data analysis in (A, B). One Way ANOVA was used for data analysis in(C). *P < .05; **P < .01; ***P < .001, respectively

**FIGURE 5:**
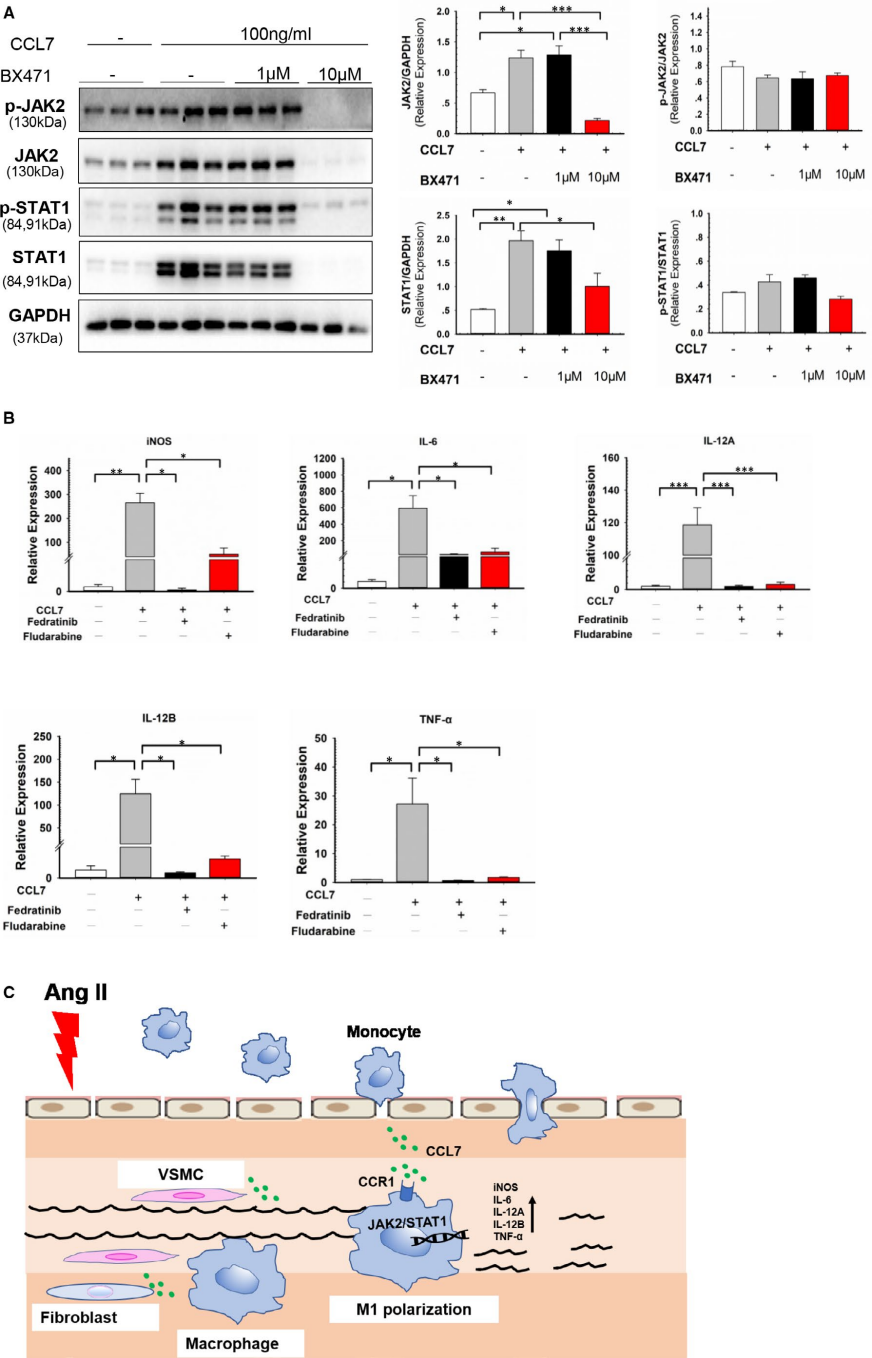
CCL7‐CCR1 interaction regulated macrophage phenotype through JAK2 /STAT1 pathway. (A) Protein abundance of JAK2, p‐JAK2, STAT1, p‐STAT1 and p‐JAK2/JAK2, p‐STAT1/STAT1 ratio in BMDMs incubated with rmCCL7 (100 ng/ml) and pre‐treated 1 hour with CCR1 antagonist‐BX471(1 μM and 10 μM). (B) M1 markers (iNOS, IL‐6, IL‐12A, IL‐12B, TNF‐α) mRNA level in BMDMs incubated with rmCCL7 alone or in combination with JAK2 inhibitor‐Fedratinib (1 μM) and STAT1 inhibitor‐Fludarabine (1 μM). (C) Pathological mechanism diagram of CCL7 in Ang II‐induced AAA. Values were represented as mean ± SEM; One Way ANOVA was used for data analysis in (A‐B) *P < .05; **P < .01; ***P < .001, respectively

JAK/STAT signalling pathway is involved in M1/M2 polarization.^9^ Our in vivo experiment showed that both JAK2 and STAT1 were up‐regulated in Ang II‐infused aortic tissues compared to control group (Figure 4A). To testify whether CCL7 contributed to JAK2/STAT1 activation, BMDMs were incubated with rmCCL7. CCL7 stimulation enhanced the expression of both total and phosphorylated JAK2/STAT1 in BMDMs (Figure 4B). Additionally, pre‐treatment of JAK2 inhibitor‐ Fedratinib eliminated the effect of CCL7 on total and phosphorylated STAT1 (Figure 4C). Meanwhile, blockage of CCR1 by BX471 inhibited CCL7‐induced JAK2/STAT1 activation (Figure 5A), indicating CCL7 activated JAK2/STAT1 pathway via binding to CCR1. To investigated whether CCL7‐CCR1 interaction regulated macrophage phenotype through JAK2/STAT1 pathway, BMDMs were pre‐treated with either Fedratinib or STAT1 inhibitor (Fludarabine). Administration of these inhibitors markedly reversed CCL7‐induced elevation of iNOS, IL‐6, IL‐12A, IL‐12B and TNF‐α mRNA level in BMDMs (Figure 5B), but had no effect on CCR1 expression (Figure S3). These findings indicated that CCL7‐CCR1 interaction promoted M1 polarization through JAK2/STAT1 signalling pathway.

To further identify the potential cell origin of CCL7, we incubated primary VSMCs, adventitial fibroblasts, BMDMs and human umbilical vein endothelial cells (HUVECs) with either Ang II or TNF‐α which could simulate the inflammatory environment in Ang II‐induced AAA,^27^, ^28^ Ang II incubation did not alter CCL7 mRNA expression in the cultured cells. There were no effects of CCL7 expression in both HUVEC and BMDMs incubated with TNF‐α as well. However, CCL7 expressions were significantly elevated in primary VSMC and adventitial fibroblast induced by TNF‐α incubation (Figure S4). The data suggested that CCL7 might be originated from medical VSMCs and adventitial fibroblasts.

We speculated that CCL7, originated from medial VSMCs and adventitial fibroblasts, could recruit macrophage accumulation in the media, activate JAK2/ STAT1 signalling by CCR1 and then promote macrophage transformation towards M1 phenotype, contributing to Ang II‐induced AAA development (Figure 4C).

### 3.4 CCL7 neutralization attenuated Ang II‐induced AAA formation

**FIGURE 6:**
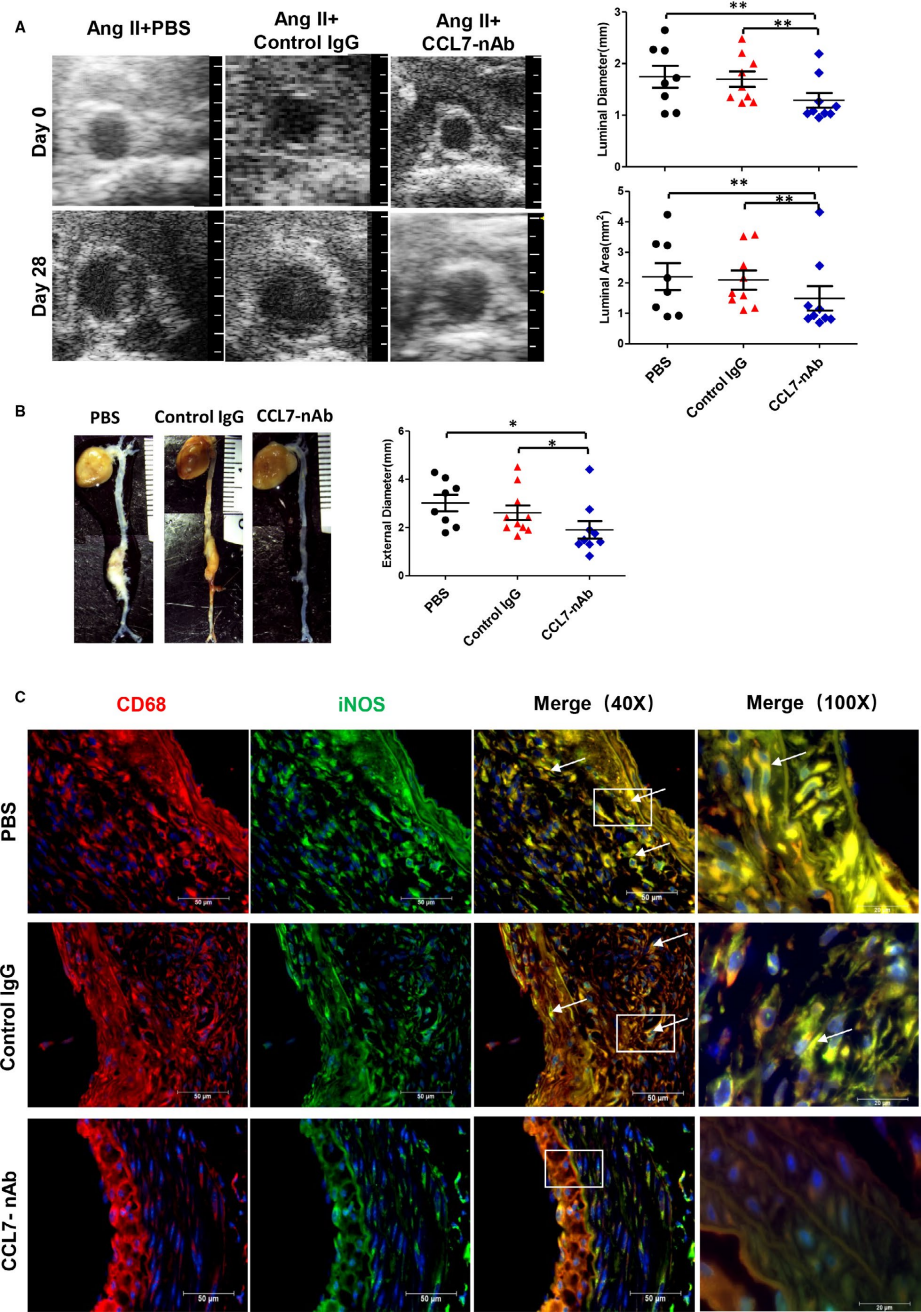
CCL7 blockade attenuated Ang II‐induced AAA. (A) Representative images of abdominal aortas measured by ultrasound including maximum abdominal aorta diameter (luminal diameter) and maximum abdominal aorta area (luminal area) of PBS (n = 10), Control IgG (n = 10) and CCL7‐nAb group (n = 10). A grid on the scale represented 0.5 mm. (B) Ex vivo maximum abdominal aorta diameter (external diameter) of PBS, Control IgG and CCL7‐nAb group. A grid on the scale represented 1 mm. (C) Representative images of M1 macrophage infiltration in abdominal aortas of PBS (n = 10), Control IgG (n = 10) and CCL7‐nAb group (n = 10) as revealed by CD68 and iNOS Immunofluorescence co‐staining. Scale bars represented 50 μm. Each plot represented 3 independent experiments. Values were represented as mean ±SEM; One Way ANOVA was used for data analysis in (A‐B). *P < .05; **P < .01; ***P < .001, respectively. White arrows (C) indicated CD68 and iNOS‐positive location

We next testified the role of CCL7 in AAA formation. Male *ApoE* ^‐/‐^ mice infused with Ang II were intraperitoneally injected with PBS, control IgG or CCL7‐neutralizing antibody (CCL7‐nAb). During the experiment, 2 mice in PBS (20%, 2/10), 1 mouse in control IgG (10%, 1/10) and 1 mouse in CCL7‐nAb (10%, 1/10) group were died of abdominal aortic rupture, no indication of thoracic aortic rupture was found in these groups. There was no statistical difference in body weight and mortality among these 3 groups (Figure S5A,B). Intriguingly, CCL7‐nAb administration significantly attenuated the dilatation of both suprarenal aortic diameter (1.29 ± 0.05 mm vs 1.74 ± 0.07 mm in PBS group and 1.70 ± 0.05 mm in control IgG group, *P* < .01) and suprarenal aortic area (1.49 ± 0.13 mm^2^ vs 2.20 ± 0.16 mm^2^ in PBS group and 2.09 ± 0.11 mm^2^ in control IgG group; *P* < .01) as revealed by ultrasound analysis at termination (Figure 6A). Consistently, blockade of CCL7 also reduced ex vivo suprarenal aortic width compared to control groups (1.91 ± 0.12 mm vs 3.02 ± 0.12 mm in PBS group and 2.61 ± 0.09 mm in control IgG group; *P* < .05; Figure 6B). Based on AAA being defined as > 50% increase in suprarenal aorta width, the AAA incidence in CCL7‐nAb group was significantly lower than control groups (30% vs 80% in PBS and control IgG group; *P* < .05). SBP was measured during Ang II infusion, which was not affected by CCL7 inhibition (Figure S5C). Collectively, our data showed that blockade of CCL7 had a strong effect on AAA retardation in a blood pressure‐independent way.

Then, we evaluated the histological alterations of abdominal aortas. Verhoeff's van Gieson and Masson staining were performed using tissue sections from suprarenal aorta to quantify the collagen and elastin content, respectively. Pronounced elastin fragmentation and collagen deposition were observed in PBS and control IgG injected mice, which was markedly reversed by CCL7 inhibition (Figure S5D). Immunofluorescence co‐staining of CD68 and iNOS revealed abundant M1 macrophages accumulation in the medial and adventitial layers in control groups while blockade of CCL7 ablated M1 macrophages from the aortic wall (Figure 6D).

## 4 DISCUSSION

The process of AAA involves a series of complex pathologies. Several mice models have been developed to simulate this pathogenesis. Among these models, Ang II‐induced AAA in mice shares many common characteristics with human AAA, such as the degradation of extracellular matrix, loss of vascular smooth muscle cells and inflammatory cells infiltration.^29^ Most importantly, the enhanced propensity for the AAA development in male mice mimics human AAA.^14^, ^30^ Male gender is a major risk factor for AAA development, the incidence rate of AAA in male is 4‐6 times that of female.^31^, ^32^ Similarly, there is a prominent gender preference in Ang II‐induced AAA (n_male_ : n_female_ ≈ 4:1).^14^, ^31^ Considering this remarkable tendency of AAA in male gender, in our study, we created AAA in male ApoE^‐/‐^ mice with Ang II infusion.

It is well documented that overactivation of inflammation is an important driving force in the pathogenesis of AAA. In this process, chemokines mediate the infiltration of multiple inflammatory cells and evoke the inflammatory cascade in the vasculature. CCL7 is initially found in human osteosarcoma cells with potent chemotaxis of leukocytes.^33^ Afterwards, numerous studies have confirmed the crucial role of CCL7 in inflammatory diseases, including infection, atherosclerosis and cancer.^34^ However, its involvement in AAA is unknown. In the present study, we found that inhibition of CCL7 profoundly attenuated AAA formation, defining CCL7 as a key participant as well as a promising therapeutic target in AAA.

Macrophage is the most predominant inflammatory component in AAA lesion. Its importance is recognized throughout the initiation, progression and healing of aneurysmal process.^3^ Growing evidence suggests the infiltration of macrophage starts from the adventitia and expands inward towards the intima.^35^ In agreement, we also revealed that macrophages were chiefly distributed in the adventitial layer in the Ang II‐induced AAA mouse model. CCL7 plays a pivotal role in macrophage recruitment.^7^, ^36^, ^37^ Our data showed inhibition of CCL7 by neutralizing antibody strikingly reduced macrophage accumulation in aortic wall. Thus, we speculate the pathogenic effect of CCL7 on AAA is achieved through attracting macrophage infiltration. The chemotaxis of CCL7 is based on its interaction with CCRs.^38^, ^39^ It is reported that CCR1, CCR2, CCR3 and CCR5 are involved in CCL7‐mediated macrophage migration and contribute to various inflammatory diseases and cancer.^7^, ^40^, ^41^, ^42^ Inhibition of CCR1, CCR2 and CCR5 has been validated to attenuate Ang II‐induced mononuclear recruitment at arterioles and venules.^43^ However, the crosstalk of CCL7/CCRs in AAA is not fully understood. In our study, CCR1 was increased in Ang II‐induced AAA tissues, but other CCR members were not changed, suggesting the pathogenic effects in aneurysmal process was predominantly executed by CCR1. Additionally, our ex vivo data showed that the antagonist of CCR1 significantly suppressed CCL7‐induced adhesion molecule activation and macrophage mobilization. These findings provided evidence that CCL7 promote AAA mainly by interacting with CCR1.

There is a vast array of macrophage subsets with diverse functions, in which M1 and M2 play a major role in AAA formation. M1 macrophages are pro‐inflammatory while M2 macrophages are anti‐inflammatory.^44^ The M1/M2 ratio determines the development of AAA.^25^ Whether CCL7 contributes to macrophage polarization is still to be delineated. In this study, we found that CCL7 promoted the pro‐inflammatory differentiation of macrophages with evidence showing enhanced M1 markers (iNOS, IL‐6, IL‐12A, IL‐12B and TNF‐α) in response to CCL7 stimulation. Cytokine signalling dependent on JAK/STAT has great impact on vascular inflammation and remodelling.^45^, ^46^ Massive studies have verified the critical role of JAK2 pathway in AAA that blocking JAK2 attenuates experimental AAA formation.^46^, ^47^ STAT family, the downstream of JAK2, is essential for macrophage polarization. It is widely known that STAT1 is crucial for M1 polarization while STAT6 is involved in M2 polarization.^9^ Activation of JAK2/STAT1 pathway has been demonstrated to contribute to various inflammatory diseases through leading macrophage towards M1 phenotype.^9^, ^48^, ^49^, ^50^, ^51^ In our ex vivo experiment, we defined CCL7 as a critical regulator of JAK2/STAT1 pathway in macrophages. Furthermore, CCL7 triggered M1 polarization was relied on JAK2/STAT1 activation. We also validated that CCL7 provoked JAK2/STAT1 through interaction with CCR1.

In summary, the present study demonstrated that CCL7 promoted macrophage infiltration and transformation towards M1 phenotype by binding to CCR1 and activating JAK2/STAT1 pathway, thus contributing to AAA formation.

## 5 CONFLICT INTERESTS

The authors declare they have no conflict of interest.

## AUTHORS CONTRIBUTION

**Cuiping Xie:** Data curation (lead); Formal analysis (lead); Project administration (lead); Writing‐original draft (lead). **Feiming Ye:** Conceptualization (supporting); Methodology (supporting); Writing‐review & editing (supporting). **Ning Zhang:** Data curation (supporting); Formal analysis (supporting); Methodology (supporting). **Yuxue Huang:** Data curation (supporting); Formal analysis (supporting); Methodology (supporting). **Yun Pan:** Conceptualization (supporting); Investigation (supporting); Supervision (supporting). **Xiaojie Xie:** Conceptualization (lead); Funding acquisition (lead); Investigation (supporting); Supervision (lead); Writing‐review & editing (lead).

## Supporting information

Supplementary Material

## Data Availability

All data are contained within the manuscript.

## References

[1] Van der Vliet JA , Boll AP . Abdominal aortic aneurysm. The Lancet. 1997;349(9055):863‐866. 10.1016/s0140-6736(96)07282-0 9121274

[2] Nordon IM , Hinchliffe RJ , Loftus IM , et al. Pathophysiology and epidemiology of abdominal aortic aneurysms. Nat Rev Cardiol. 2011;8(2):92‐102. 21079638 10.1038/nrcardio.2010.180

[3] Raffort J , Lareyre F , Clément M , et al. Monocytes and macrophages in abdominal aortic aneurysm. Nat Rev Cardiol. 2017;14(8):457‐471. 28406184 10.1038/nrcardio.2017.52

[4] Murray PJ . Macrophage polarization. Annu Rev Physiol. 2017;79:541‐566. 27813830 10.1146/annurev-physiol-022516-034339

[5] Zouggari Y , Ait‐Oufella H , Bonnin P , et al. B lymphocytes trigger monocyte mobilization and impair heart function after acute myocardial infarction. Nat Med. 2013;19(10):1273‐1280. 24037091 10.1038/nm.3284 PMC4042928

[6] Lee PY , Li Y , Kumagai Y , et al. Type I interferon modulates monocyte recruitment and maturation in chronic inflammation. The American journal of pathology. 2009;175(5):2023‐2033. 19808647 10.2353/ajpath.2009.090328 PMC2774066

[7] Tsou CL , Peters W , Si Y , et al. Critical roles for CCR2 and MCP‐3 in monocyte mobilization from bone marrow and recruitment to inflammatory sites. J Clin Invest. 2007;117(4):902‐909. 17364026 10.1172/JCI29919 PMC1810572

[8] Huang J , Zhang Z , Guo J , et al. Genetic modification of mesenchymal stem cells overexpressing CCR1 increases cell viability, migration, engraftment, and capillary density in the injured myocardium. Circ Res. 2010;106(11):1753‐1762. 20378860 10.1161/CIRCRESAHA.109.196030 PMC2884066

[9] Lawrence T , Natoli G . Transcriptional regulation of macrophage polarization: Enabling diversity with identity. Nat Rev Immunol. 2011;11:750‐761. 22025054 10.1038/nri3088

[10] Akram M , Kim KA , Kim ES , et al. Selective inhibition of JAK2/STAT1 signaling and iNOS expression mediates the anti‐inflammatory effects of coniferyl aldehyde. Chem Biol Interact. 2016;256:102‐110. 27378624 10.1016/j.cbi.2016.06.029

[11] Tsoyi K , Kim HJ , Shin JS , et al. HO‐1 and JAK‐2/STAT‐1 signals are involved in preferential inhibition of iNOS over COX‐2 gene expression by newly synthesized tetrahydroisoquinoline alkaloid, CKD712, in cells activated with lipopolysacchride. Cell Signal. 2008;20(10):1839‐1847. 18634870 10.1016/j.cellsig.2008.06.012

[12] Darnell JE Jr , Kerr IM , Stark GR . Jak‐STAT pathways and transcriptional activation in response to IFNs and other extracellular signaling proteins. Science. 1994;264(5164):1415‐1421. 8197455 10.1126/science.8197455

[13] Daugherty A , Rateri D , Lu H , et al. Measuring blood pressure in mice using volume pressure recording, a tail‐cuff method. J Vis Exp. 2009;27:1291. 10.3791/1291 PMC2794298 19488026

[14] Daugherty A , Cassis LA , Lu H . Complex pathologies of angiotensin II‐induced abdominal aortic aneurysms. J Zhejiang Univ Sci B. 2011;12(8):624‐628. 21796801 10.1631/jzus.B1101002 PMC3150714

[15] Wang Q , Ding Y , Song P , et al. Tryptophan‐Derived 3‐Hydroxyanthranilic Acid Contributes to Angiotensin II‐Induced Abdominal Aortic Aneurysm Formation in Mice In Vivo. Circulation. 2017;136(23):2271‐2283. 28978552 10.1161/CIRCULATIONAHA.117.030972 PMC5716872

[16] Westermann D , Savvatis K , Lindner D , et al. Reduced degradation of the chemokine MCP‐3 by matrix metalloproteinase‐2 exacerbates myocardial inflammation in experimental viral cardiomyopathy. Circulation. 2011;124(19):2082‐2093. 21986287 10.1161/CIRCULATIONAHA.111.035964

[17] Qi D , Wei M , Jiao S , et al. Hypoxia inducible factor 1alpha in vascular smooth muscle cells promotes angiotensin II‐induced vascular remodeling via activation of CCL7‐mediated macrophage recruitment. Cell Death Dis. 2019;10(8):544. 31320613 10.1038/s41419-019-1757-0 PMC6639417

[18] Barisione C , Charnigo R , Howatt DA , et al. Rapid dilation of the abdominal aorta during infusion of angiotensin II detected by noninvasive high‐frequency ultrasonography. J Vasc Surg. 2006;44:372‐376. 16890871 10.1016/j.jvs.2006.04.047

[19] Daugherty A , Manning MW , Cassis LA . Angiotensin II promotes atherosclerotic lesions and aneurysms in apolipoprotein e‐deficient mice. J Clin Invest. 2000;105(11):1605‐1612. 10841519 10.1172/JCI7818 PMC300846

[20] Chang HR , Josefs T , Scerbo D , et al. Role of LpL (Lipoprotein Lipase) in Macrophage Polarization In Vitro and In Vivo. Arterioscler Thromb Vasc Biol. 2019;39(10):1967‐1985. 31434492 10.1161/ATVBAHA.119.312389 PMC6761022

[21] Park YM , Febbraio M . CD36 modulates migration of mouse and human macrophages in response to oxidized LDL and may contribute to macrophage trapping in the arterial intima. Journal of Clinical Investigation. J Clin Invest. 2009; 119(1):136–145. 10.1172/JCI35535 PMC2613464 19065049

[22] Wang Q , Liu Z , Ren J , Morgan S , Assa C , Liu B . Receptor‐interacting protein kinase 3 contributes to abdominal aortic aneurysms via smooth muscle cell necrosis and inflammation. Circ Res. 2015;116(4):600‐611. 25563840 10.1161/CIRCRESAHA.116.304899 PMC4329096

[23] Li Y , Tao J , Zhang J , et al. Cellular repressor e1a‐stimulated genes controls phenotypic switching of adventitial fibroblasts by blocking p38mapk activation. Atherosclerosis. 2012;225(2):304‐314. 23040447 10.1016/j.atherosclerosis.2012.08.015

[24] Cheng JW , Sadeghi Z , Levine AD , et al. The role of CXCL12 and CCL7 chemokines in immune regulation, embryonic development, and tissue regeneration. Cytokine. 2014;69(2):277‐283. 25034237 10.1016/j.cyto.2014.06.007

[25] Qin Z , Bagley J , Sukhova G , et al. Angiotensin II‐induced TLR4 mediated abdominal aortic aneurysm in apolipoprotein E knockout mice is dependent on STAT3. J Mol Cell Cardiol. 2015;87:160‐170. 26299839 10.1016/j.yjmcc.2015.08.014

[26] Maddaluno M , Di Lauro M , Di Pascale A , et al. Monocyte chemotactic protein‐3 induces human coronary smooth muscle cell proliferation. Atherosclerosis. 2011;217(1):113‐119. 21536288 10.1016/j.atherosclerosis.2011.04.002

[27] Liang ES , Bai WW , Wang H , et al. PARP‐1 (Poly[ADP‐Ribose] Polymerase 1) inhibition protects from Ang II (angiotensin II)‐induced abdominal aortic aneurysm in mice. Hypertension. 2018;72(5):1189‐1199. 30354818 10.1161/HYPERTENSIONAHA.118.11184

[28] Chiang MT , Chen IM , Hsu FF , et al. Gal‐1 (galectin‐1) upregulation contributes to abdominal aortic aneurysm progression by enhancing vascular inflammation. Arterioscler Thromb Vasc Biol. 2021;41(1):331‐345. 33147994 10.1161/ATVBAHA.120.315398

[29] Sakalihasan N , Michel JB , Katsargyris A , et al. Abdominal aortic aneurysms. Nature reviews. Disease primers. 2018;4(1):34. 10.1038/s41572-018-0030-7 30337540

[30] Daugherty A , Cassis LA . Mouse models of abdominal aortic aneurysms. Arterioscler Thromb Vasc Biol. 2004;24(3):429‐434. 14739119 10.1161/01.ATV.0000118013.72016.ea

[31] Robinet P , Milewicz DM , Cassis LA , et al. Consideration of Sex Differences in Design and Reporting of Experimental Arterial Pathology Studies‐Statement From ATVB Council. Arterioscler Thromb Vasc Biol. 2018;38(2):292‐303. 29301789 10.1161/ATVBAHA.117.309524 PMC5785439

[32] Villard C , Hultgren R . Abdominal aortic aneurysm: Sex differences. Maturitas. 2018;109:63‐69. 29452784 10.1016/j.maturitas.2017.12.012

[33] Van Damme J , Proost P , Lenaerts JP , et al. Structural and functional identification of two human, tumor‐derived monocyte chemotactic proteins (MCP‐2 and MCP‐3) belonging to the chemokine family. J Exp Med. 1992;176(1):59–65. 1613466 10.1084/jem.176.1.59 PMC2119277

[34] Ford J , Hughson A , Lim K , et al. CCL7 Is a Negative Regulator of Cutaneous Inflammation Following Leishmania major Infection. Front Immunol. 2018;9:3063. 30671055 10.3389/fimmu.2018.03063 PMC6331479

[35] Maiellaro K , Taylor WR . The role of the adventitia in vascular inflammation. Cardiovasc Res. 2007;75(4):640‐648. 17662969 10.1016/j.cardiores.2007.06.023 PMC3263364

[36] Su B , Zhao W , Shi B , et al. Let‐7d suppresses growth, metastasis, and tumor macrophage infiltration in renal cell carcinoma by targeting COL3a1 and CCL7. Mol Cancer. 2014;13:206. 25193015 10.1186/1476-4598-13-206 PMC4168121

[37] He J , Song Y , Li G , et al. Fbxw7 increases CCL2/7 in CX3CR1hi macrophages to promote intestinal inflammation. J Clin Invest. 2019;129(9):3877‐3893. 31246581 10.1172/JCI123374 PMC6715366

[38] Zlotnik A , Yoshie O , Nomiyama H . The chemokine and chemokine receptor superfamilies and their molecular evolution. Genome Biol. 2006;7(12):243. 17201934 10.1186/gb-2006-7-12-243 PMC1794421

[39] Zlotnik A , Yoshie O . The chemokine superfamily revisited. Immunity. 2012;36(5):705‐716. 22633458 10.1016/j.immuni.2012.05.008 PMC3396424

[40] Wilson GJ , Fukuoka A , Love SR , et al. Chemokine receptors coordinately regulate macrophage dynamics and mammary gland development. Development. 2020;147(12):dev187815. 32467242 10.1242/dev.187815 PMC7328164

[41] Korbecki J , Kojder K , Barczak K , et al. Hypoxia Alters the Expression of CC Chemokines and CC Chemokine Receptors in a Tumor‐A Literature Review. Int J Mol Sci. 2020;21(16):5647. 10.3390/ijms21165647 PMC7460668 32781743

[42] Huber J , Kiefer FW , Zeyda M , et al. CC chemokine and CC chemokine receptor profiles in visceral and subcutaneous adipose tissue are altered in human obesity. J Clin Endocrinol Metab. 2008;93(8):3215‐3221. 18492752 10.1210/jc.2007-2630

[43] Mateo T , Naim Abu Nabah Y , Abu Taha M , et al. Angiotensin II‐induced mononuclear leukocyte interactions with arteriolar and venular endothelium are mediated by the release of different CC chemokines. J Immunol. 2006;176(9):5577‐5586. 16622027 10.4049/jimmunol.176.9.5577

[44] Murray PJ , Wynn TA . Protective and pathogenic functions of macrophage subsets. Nat Rev Immunol. 2011;11(11):723‐737. 21997792 10.1038/nri3073 PMC3422549

[45] Ivashkiv LB . IFNgamma: Signalling, epigenetics and roles in immunity, metabolism, disease and cancer immunotherapy. Nat Rev Immunol. 2018;18(9):545‐558. 29921905 10.1038/s41577-018-0029-z PMC6340644

[46] Xiao J , Wei Z , Chen X , et al. Experimental abdominal aortic aneurysm growth is inhibited by blocking the JAK2/STAT3 pathway. Int J Cardiol. 2020;312:100‐106. 32334849 10.1016/j.ijcard.2020.03.072

[47] Tanaka T , Kelly M , Takei Y , et al. RANKL‐mediated osteoclastogenic differentiation of macrophages in the abdominal aorta of angiotensin II‐infused apolipoprotein E knockout mice. J Vasc Surg. 2018;68(6):48S‐59S.. e1 10.1016/j.jvs.2017.11.091 PMC6558660 29685509

[48] Zhao M , Bian YY , Yang LL , et al. Huoxuetongfu formula alleviates intraperitoneal adhesion by regulating macrophage polarization and the SOCS/JAK2/STAT/PPAR‐gamma signalling pathway. Mediators Inflamm. 2019;2019:1769374. 31772499 10.1155/2019/1769374 PMC6854253

[49] Oh H , Park SH , Kang MK , et al. Asaronic Acid Attenuates Macrophage Activation toward M1 Phenotype through Inhibition of NF‐κB Pathway and JAK‐STAT Signaling in Glucose‐Loaded Murine Macrophages. Journal of agricultural and food chemistry. 2019;67(36):10069‐10078. 31422663 10.1021/acs.jafc.9b03926

[50] Liu Q , Xie W , Wang Y , et al. JAK2/STAT1‐mediated HMGB1 translocation increases inflammation and cell death in a ventilator‐induced lung injury model. Lab Invest. 2019;99(12):1810‐1821. 31467427 10.1038/s41374-019-0308-8

[51] Xi J , Huang Q , Wang L , et al. miR‐21 depletion in macrophages promotes tumoricidal polarization and enhances PD‐1 immunotherapy. Oncogene. 2018;37(23):3151‐3165. 29540832 10.1038/s41388-018-0178-3 PMC5993583

