# Supplementary material for: CCL7 contributes to angiotensin II‐induced abdominal aortic aneurysm by promoting macrophage infiltration and pro‐inflammatory phenotype
Source: J Cell Mol Med. 2021 Jun 29;25(15):7280–93. doi: 10.1111/jcmm.16757 (PMC8335673; doi:10.1111/jcmm.16757)
Supplement: Supplementary file 1 — Supplementary Material [file JCMM-25-7280-s001.pptx]

## Slide 1
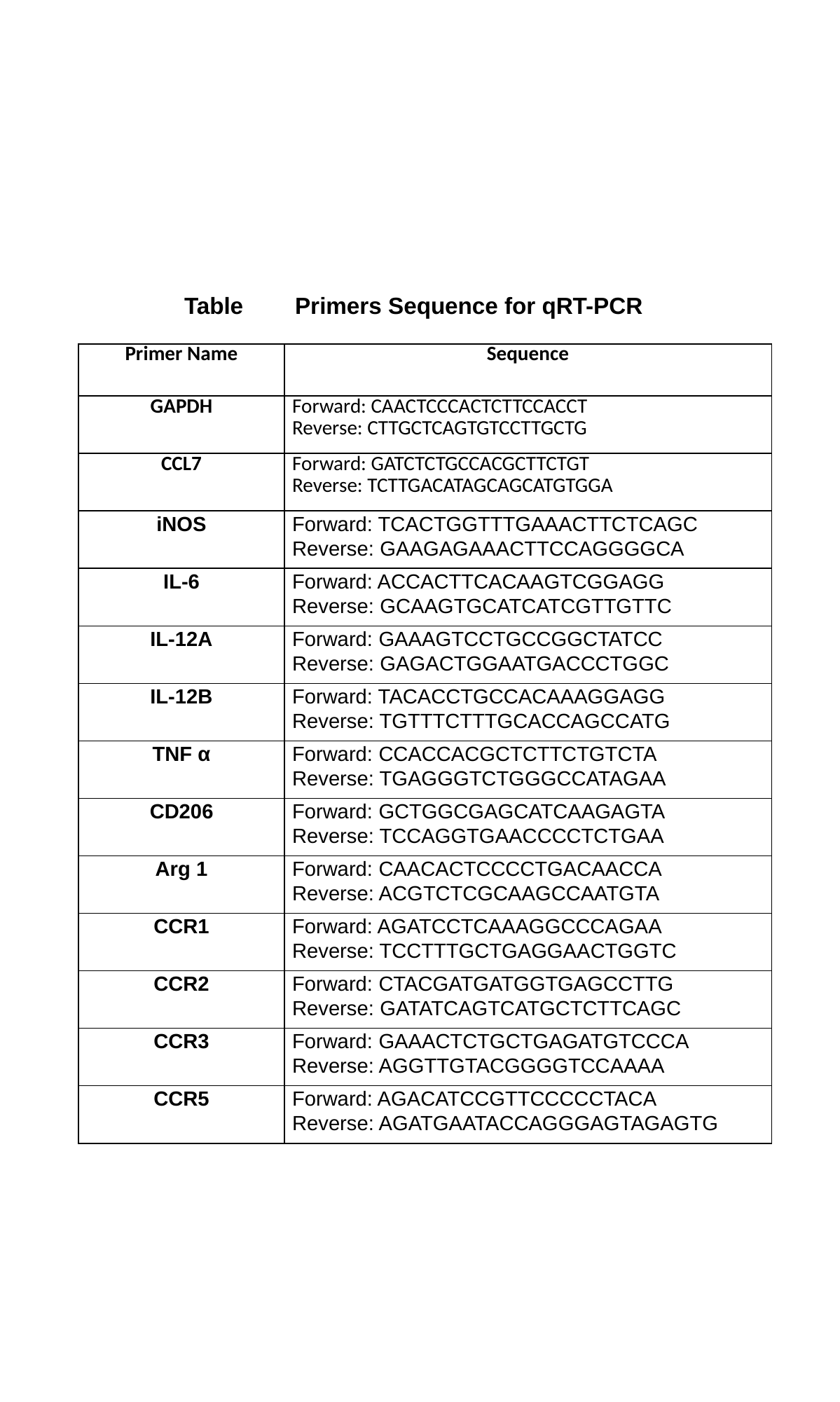

Table Primers Sequence for qRT-PCR
| Primer Name | Sequence |
| --- | --- |
| GAPDH | Forward: CAACTCCCACTCTTCCACCT Reverse: CTTGCTCAGTGTCCTTGCTG |
| CCL7 | Forward: GATCTCTGCCACGCTTCTGT Reverse: TCTTGACATAGCAGCATGTGGA |
| iNOS | Forward: TCACTGGTTTGAAACTTCTCAGC Reverse: GAAGAGAAACTTCCAGGGGCA |
| IL-6 | Forward: ACCACTTCACAAGTCGGAGG Reverse: GCAAGTGCATCATCGTTGTTC |
| IL-12A | Forward: GAAAGTCCTGCCGGCTATCC Reverse: GAGACTGGAATGACCCTGGC |
| IL-12B | Forward: TACACCTGCCACAAAGGAGG Reverse: TGTTTCTTTGCACCAGCCATG |
| TNF α | Forward: CCACCACGCTCTTCTGTCTA Reverse: TGAGGGTCTGGGCCATAGAA |
| CD206 | Forward: GCTGGCGAGCATCAAGAGTA Reverse: TCCAGGTGAACCCCTCTGAA |
| Arg 1 | Forward: CAACACTCCCCTGACAACCA Reverse: ACGTCTCGCAAGCCAATGTA |
| CCR1 | Forward: AGATCCTCAAAGGCCCAGAA Reverse: TCCTTTGCTGAGGAACTGGTC |
| CCR2 | Forward: CTACGATGATGGTGAGCCTTG Reverse: GATATCAGTCATGCTCTTCAGC |
| CCR3 | Forward: GAAACTCTGCTGAGATGTCCCA Reverse: AGGTTGTACGGGGTCCAAAA |
| CCR5 | Forward: AGACATCCGTTCCCCCTACA Reverse: AGATGAATACCAGGGAGTAGAGTG |

## Slide 2
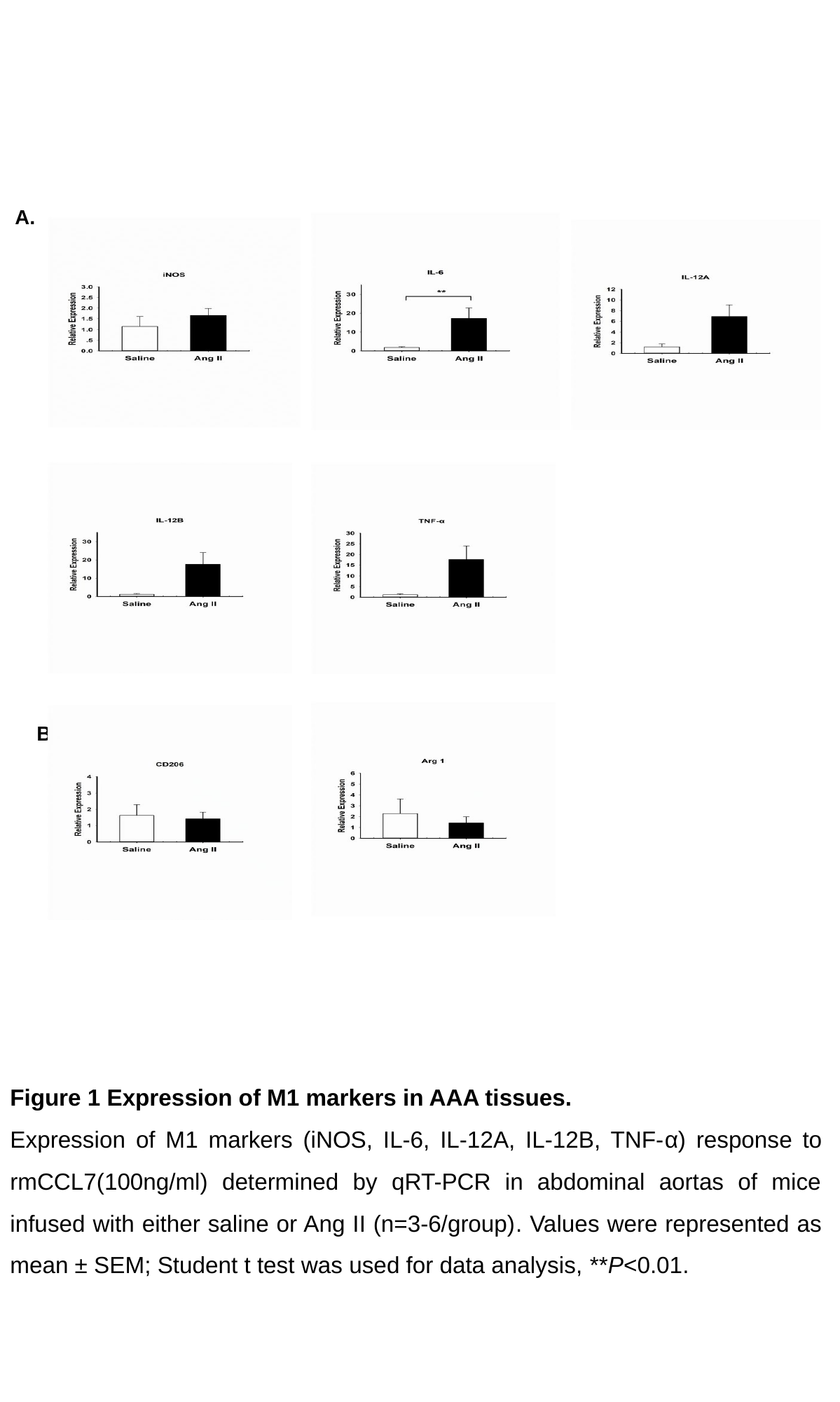

A.
B.
Figure 1 Expression of M1 markers in AAA tissues.
Expression of M1 markers (iNOS, IL-6, IL-12A, IL-12B, TNF-α) response to rmCCL7(100ng/ml) determined by qRT-PCR in abdominal aortas of mice infused with either saline or Ang II (n=3-6/group). Values were represented as mean ± SEM; Student t test was used for data analysis, **P<0.01.

## Slide 3
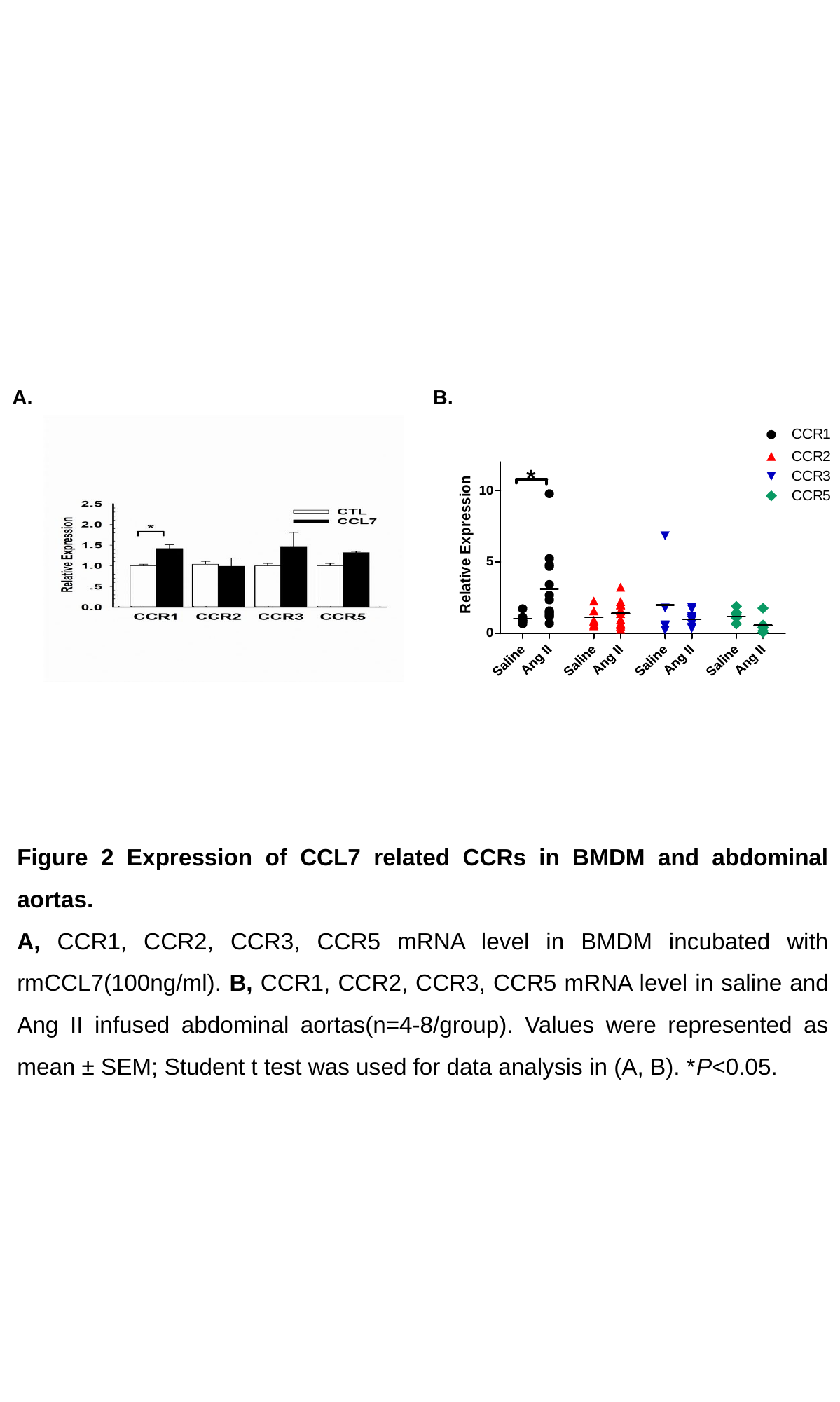

A.
B.
Figure 2 Expression of CCL7 related CCRs in BMDM and abdominal aortas.
A, CCR1, CCR2, CCR3, CCR5 mRNA level in BMDM incubated with rmCCL7(100ng/ml). B, CCR1, CCR2, CCR3, CCR5 mRNA level in saline and Ang II infused abdominal aortas(n=4-8/group). Values were represented as mean ± SEM; Student t test was used for data analysis in (A, B). *P<0.05.

## Slide 4
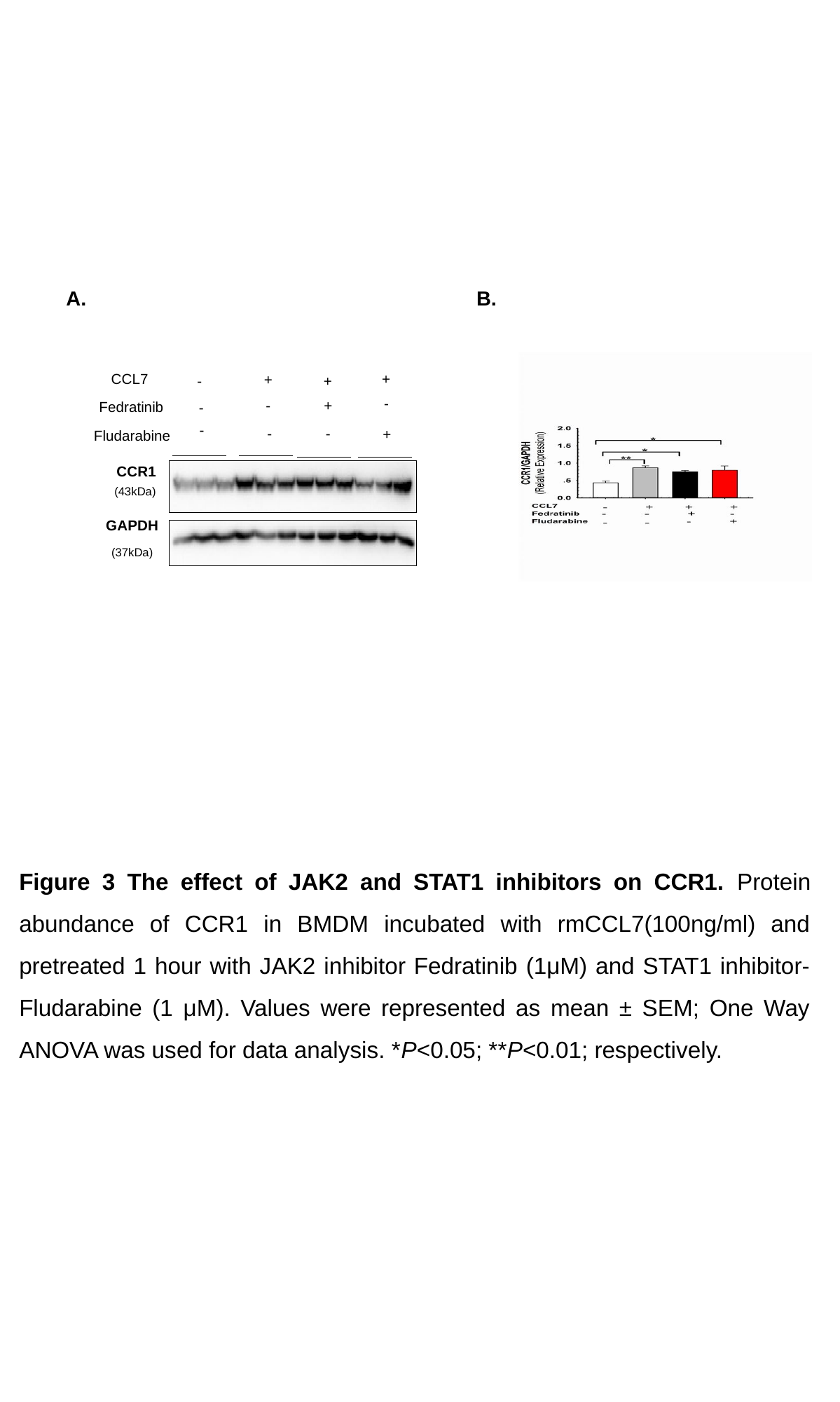

B.
A.
CCL7
+
+
+
-
-
-
+
Fedratinib
-
-
-
-
+
Fludarabine
CCR1
(43kDa)
GAPDH
(37kDa)
Figure 3 The effect of JAK2 and STAT1 inhibitors on CCR1. Protein abundance of CCR1 in BMDM incubated with rmCCL7(100ng/ml) and pretreated 1 hour with JAK2 inhibitor Fedratinib (1μM) and STAT1 inhibitor-Fludarabine (1 μM). Values were represented as mean ± SEM; One Way ANOVA was used for data analysis. *P<0.05; **P<0.01; respectively.

## Slide 5
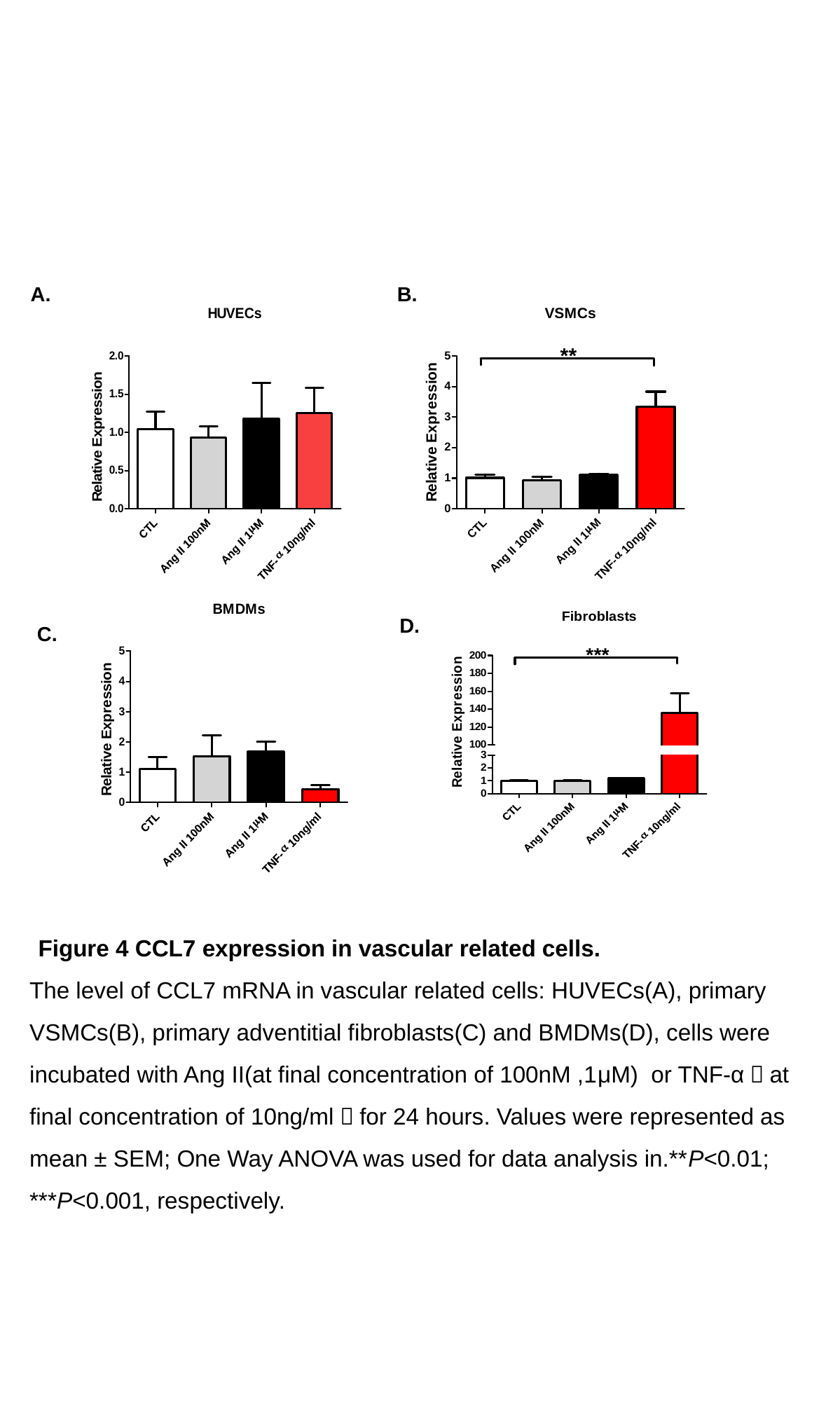

A.
B.
D.
C.
Figure 4 CCL7 expression in vascular related cells.
The level of CCL7 mRNA in vascular related cells: HUVECs(A), primary VSMCs(B), primary adventitial fibroblasts(C) and BMDMs(D), cells were incubated with Ang II(at final concentration of 100nM ,1μM) or TNF-α（at final concentration of 10ng/ml）for 24 hours. Values were represented as mean ± SEM; One Way ANOVA was used for data analysis in.**P<0.01; ***P<0.001, respectively.

## Slide 6
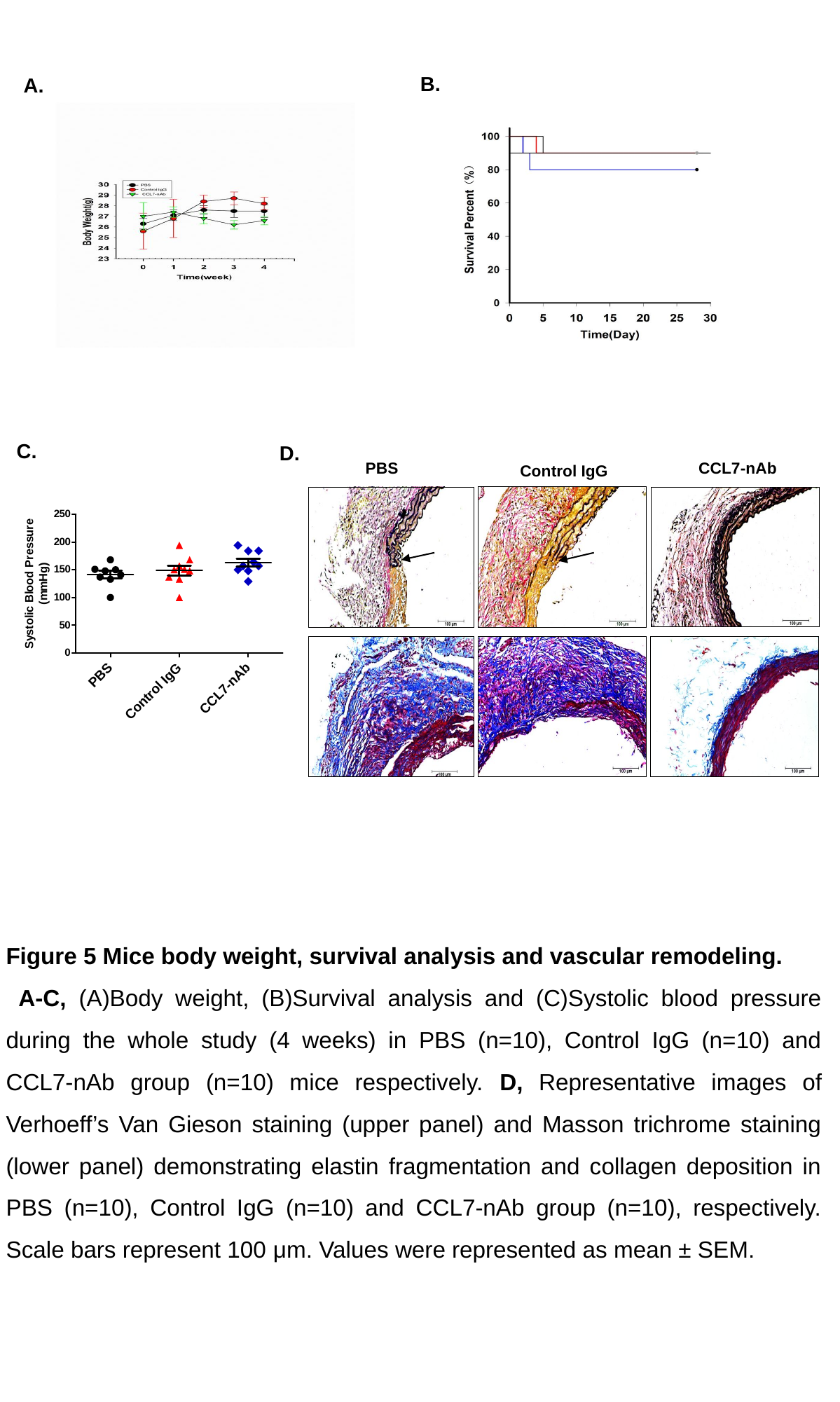

B.
A.
C.
D.
PBS
CCL7-nAb
Control IgG
Figure 5 Mice body weight, survival analysis and vascular remodeling.
 A-C, (A)Body weight, (B)Survival analysis and (C)Systolic blood pressure during the whole study (4 weeks) in PBS (n=10), Control IgG (n=10) and CCL7-nAb group (n=10) mice respectively. D, Representative images of Verhoeff’s Van Gieson staining (upper panel) and Masson trichrome staining (lower panel) demonstrating elastin fragmentation and collagen deposition in PBS (n=10), Control IgG (n=10) and CCL7-nAb group (n=10), respectively. Scale bars represent 100 μm. Values were represented as mean ± SEM.
